# Essentials of Physical Diagnosis of the Thorax

**Published:** 1900-03

**Authors:** 


ESSENTIALS OF PHYSICAL DIAGNOSIS OF THE THORAX. By ARTHUR M. CORWIN, A. M, M. D., Instructor of Physical Diagnosis at Rush Medical College; Attending Physician to the Central Free Dispensary, etc. Third edition, revised and enlarged. Philadelphia: W. B. Saunders. 1899.

This little work on physical diagnosis of the thorax was designed primarily by Dr. Corwin to meet the wants of his own classes. So well presented was the subject that a second and now a third edition followed. The book is an excellent one for the student, the text being so arranged that the eye at once catches the salient points which are thus easier held in memory. We commend the volume to those looking for a brief and concisely written student's manual on this subject. M. J. F.

